# Fatal Injury of the Small Intestine during Retropubic Sling Placement

**DOI:** 10.1155/2015/164545

**Published:** 2015-10-18

**Authors:** P. Kascak, B. Kopcan

**Affiliations:** ^1^Department of Obstetrics and Gynecology, General Hospital, Trenčín, Slovakia; ^2^Faculty of Health, Alexander Dubcek University, 911 01 Trenčín, Slovakia

## Abstract

We describe a case of injury of the small intestine in a patient who underwent placement of Align R retropubic urethral support system (BARD). Absence of characteristic symptoms of the bowel injury and peritonitis led to a rapid development of sepsis, multiple organ failure, and death. Although the placement of midurethral sling is a minimally invasive surgery, good diagnostic skills, proper evaluation of indications, safe performance of the procedure, and thorough postsurgical monitoring are paramount for safe and effective outcome of the surgery.

## 1. Introduction

Placement of midurethral sling became a preferred and highly effective method of surgical treatment of stress incontinence [1]. Nevertheless it is minimally invasive procedure; many studies show that it may be connected with various complications [2]. Although the most frequent injuries occur in retroperitoneum, some authors describe injuries of the organs within abdominal cavity.

## 2. Case Report

A 66-year-old female was admitted to gynecology unit for surgical treatment of stress incontinence. After urodynamic testing and clinical examination, the retropubic midurethral sling was recommended. Previously, this patient underwent 3 abdominal procedures, right nephrectomy, hysterectomy with bilateral adnexectomy, and cholecystectomy. She had history of myocarditis and was treated for hypertension, diabetes mellitus, and hypothyroidism. Patient was obese (BMI = 33) and had pendulous abdomen. The surgeon was an experienced specialist with proper training in midurethral sling placement. Surgery was done without any complications. Align R retropubic urethral support system (BARD) was used. During surgery aquadissection was not performed and cystoscopy was negative. No complications were noted in the immediate post-op observation period. On the first postoperative day patient complained of nausea and vomitus which was interpreted as side effect of general anesthesia. Blood pressure (BP) was 160/85 and 130/70, pulse rate 92 and 78 per minute, serum glucose 11.0 and 11.7 mmol/L, and urine residuum 70 mL. Patient did not complain of any pain or distress; physical examination did not show any abnormal findings. Patient was afebrile. On the second postoperative day, before discharge, patient complained that she could not urinate; 300 mL of urine was obtained with catheterisation. BP was 135/65 and serum glucose 12.0 mmol/L. She was denying any other difficulties or abdominal pain. She was afebrile; abdomen was soft and nontender. In the afternoon, in spite of increased oral fluids intake patient was still oliguric, with only 200 mL of urine output. Patient was drowsy and tired. Laboratory results showed elevated urea at 25.1 mmol/L and creatinine 223 *μ*mol/L, ionogram was normal, and glucose was 13.5 mmol/L. Patient had bowel movement; the stool was black and watery. It was suggested that patient developed melena and renal insufficiency. Patient did not complain of any pain. Consult with internal medicine specialist and gastroenterologist was performed and parenteral hydration was ordered. Patient continued to be oliguric; the abdomen was without signs and symptoms of peritoneal irritation or acute abdominal condition. Although patient was afebrile and the blood tests showed only mild leukocytosis (13.1 × 10^9^/L), CRP level was found to be significantly elevated (396.55 mg/L). Patient was started on combination of broad-spectrum antibiotics. Ultrasound of lesser pelvis and of solitary kidney did not reveal any abnormality. Urologist was consulted; however, the cause of renal insufficiency was not clear. Six hours after initial complaint of oliguria, patient started to complain of difficulty in breathing and pain in epigastric area. Vital functions were stable. Surgeon on-call examined patient but did not find any signs and symptoms of acute abdominal condition. In spite of negative clinical exam, CT of abdomen was ordered, which showed pneumoperitoneum and phlegmonous infiltration of abdominal wall. On the basis of worsening patient's condition, laboratory, and CT results, working diagnosis of acute sepsis caused by peracute phlegmonous inflammation and possible injury of gastrointestinal tract was made. Eight hours after initial complaint, patient underwent explorative laparotomy by the on-call surgical team. During surgery it was confirmed that patient had sustained injury to the loop of the small bowel in the right hypogastric area. Sling was found to be penetrating the loop of the intestine. In lesser pelvis adhesions from previous laparotomy were noted. Surgery confirmed presence of intestinal contents in abdominal cavity and widespread peritonitis. The tape was removed, lavage of abdominal cavity was performed, and perforated bowel was sutured. During the surgery patient was resuscitated two times due to cardiopulmonary arrest. After the surgery patient was transferred to the ICU and treated for septic shock. In spite of aggressive treatment, patient expired on the third day after sling placement.

## 3. Discussion

Placement of midurethral sling has been a common procedure in our department since 2003. By* May 2013*,* 642* surgeries were performed, perioperative and postoperative complications occurred in 4.8% of patients. The case of an intestinal injury during surgery had never occurred before and because of its rare incidence and atypical symptoms we were not thinking about this possibility. Meta-analysis covering the period from 1995 to 2007 showed that in retropubic method complications occur in 4.3%–75.1% of patients, and in transobturator method complications occur in 10.5%–31.3% of patients [3]. Retropubic method is associated not only with higher percentage of complications but also with more serious injuries, such as injury to the intestine or large blood vessels. Also, this method is connected with a higher risk of death [3]. The most common problem of patients with midurethral sling is difficulty in urinating [4]. In our case, on-call staff initially assumed that patient is experiencing this type of complication and therefore he ordered hydration and monitoring of urinary output. On the first postoperative day patient was not showing any signs of serious complications. Only on the second postoperative day the condition of the patient started to deteriorate. Correct diagnosis was established on the second postoperative day in the evening (56 hours after surgery). In spite of the fact that adequate and aggressive surgical and medical treatment was implemented, the patient died 72 hours after surgery. Decisive moment in establishing diagnosis was the CRP value, which was significantly elevated and led on-call staff suspect abdominal complications and sepsis. Consequently, evaluation of all abnormal findings led to conclusion that patient had sustained perioperative injury of the gastrointestinal tract.

Injury to the intestine is considered to be a rare complication. By 2004, injury to the intestine during placement of midurethral sling was reported in 35 patients out of 700 000 cases (0.005%) [5]. Seven patients from this group died. Death of 6 patients was directly related to the injury to the intestine but in 5 cases the complication was not detected before death. Agostini in his report analyzed 12 280 midurethral sling surgeries and described occurrence of injury to the intestine only in 3 patients (0.02%) [6]. Reports show that injury to the intestine occurs most frequently in older, skinny females whose past medical history includes surgery of the lesser pelvis [7, 8]. In our case, on the contrary, BMI level was high; however, other risk factors were present. Peritonitis due to injury of intestinal loop is a potentially fatal complication; in most cases it is detected shortly after the surgery [9]. Injury to the intestine is usually corrected by revision laparotomy. Meschia describes a case when the small intestine was injured during the placement of sling and 5 hours later laparoscopy was performed because patient developed symptoms of acute abdominal condition [10]. Injury was corrected successfully by endoscopic procedure. In our case signs and symptoms of peritoneal irritation or fever were not present. Cases when complications were unrecognized after the surgery are described in many studies [7]. Huffaker describes the case when perforation was detected only on the 4th postoperative day [11]. In this patient, similarly to our case, typical symptoms of acute abdominal condition development were absent. In the case study reported by Olagundoye, patient presented with various problems since the 2nd day after surgery; however, the diagnosis of intestine perforation was established only on day 7 postoperatively [5]. Emergency laparotomy was recommended; however, patient gave consent to procedure only on the 24th day after surgery. Elliot reports the case when the complication was detected only 5 years after the procedure during abdominal surgery for different reason [8]. However, if the complication development is asymptomatic, it is possible that it was caused by gradual intestine erosion rather than by direct penetrating injury of the intestine by the introducer of the tape [12]. In high risk patients retropubic midurethral sling placement should only be performed by highly experienced urogynecologist as an in-patient procedure [10].

## 4. Conclusion

Potentially fatal injury of the intestine is one of the risks of the new methods of treatment of stress incontinence. Our case study describes initially mild and later fatal course of development of complications in the patient with retropubic midurethral sling placement. Our experience shows that good diagnostic skills, careful evaluation of indications for the procedure, safe performance of the surgery, and thorough postsurgical monitoring are paramount in postoperative management of patients with stress incontinence.
